# The Quorum Sensing Inhibitor Qstatin Has Broad-Spectrum Antivirulence Activity Towards Shrimp-Pathogenic Vibrios

**DOI:** 10.3390/microorganisms14051065

**Published:** 2026-05-09

**Authors:** Peizhuo Zou, Qian Yang, Tom Defoirdt

**Affiliations:** Center for Microbial Ecology and Technology, Ghent University, Frieda Saeysstraat 1, 9052 Gent, Belgium; peizhuo.zou@ugent.be (P.Z.); qian.yang@ugent.be (Q.Y.)

**Keywords:** quorum sensing, quorum quenching, virulence factors, antivirulence therapy

## Abstract

The emergence of antibiotic resistance in aquaculture not only makes antibiotic treatments ineffective in aquaculture but also poses a threat to public health. In order to overcome this, novel strategies to control bacterial diseases are needed. Antivirulence therapy, which disrupts virulence without affecting bacterial viability, represents a promising alternative approach. This study evaluated the antivirulence activity of Qstatin against pathogenic vibrios belonging to the *Harveyi* clade. Qstatin specifically inhibited the three-channel quorum sensing system in *Vibrio campbellii*, significantly downregulated the expression of quorum sensing-regulated virulence genes (*flaA*, *flaK*, *vpsR*, *vpsT*, and *vhp*) and attenuated the corresponding phenotypes: swimming motility was reduced by up to 57% and biofilm formation by up to 76%. Protease activity, in contrast, was slightly increased rather than decreased. Finally, treatment with 100 μM Qstatin significantly increased the survival of gnotobiotic brine shrimp larvae upon challenge with each of 13 tested pathogenic *Harveyi* clade strains (belonging to the species *V. campbellii*, *V. harveyi*, or *V. parahaemolyticus*), without an impact on *Vibrio* densities in the rearing water. These findings indicate that Qstatin has a broad-spectrum antivirulence activity against *Harveyi* clade vibrios by inhibiting quorum sensing, thus supporting its potential as a sustainable disease control agent.

## 1. Introduction

Disease outbreaks are posing significant challenges for aquaculture. *Vibrio* species are highly motile Gram-negative bacteria that are ubiquitous in aquatic ecosystems [1,2]. At this moment, vibrios belonging to the *Harveyi* clade (including *V. harveyi* and closely related species, such as *V. campbellii* and *V. parahaemolyticus*) are the most significant bacterial pathogens in marine aquaculture, dramatically affecting all types of farmed aquatic animals, including crustaceans, fish, and molluscs [3,4]. In addition to aquatic organisms, some *V. parahaemolyticus* strains also cause infections in humans [5]. One of the most devastating diseases caused by *Harveyi* clade vibrios that is currently hitting shrimp aquaculture particularly hard is Acute Hepatopancreatic Necrosis Disease (AHPND). According to the World Organization for Animal Health (WOAH, formerly OIE), outbreaks of AHPND resulted in shrimp farming losses exceeding $44 billion across China, Malaysia, Mexico, Thailand, and Vietnam from 2010 to 2016 [6]. The virulence of AHPND-causing *Vibrio* spp. strains is derived from a ~70 kbp conjugative plasmid (pVA) containing the *priA* and *pirB* toxin genes, which encode a binary toxin that targets the shrimp’s hepatopancreatic cells [7]. AHPND develops rapidly, starting approximately 8 days after stocking shrimp post-larvae into ponds, and severe mortalities (up to 100%) occur within 20–30 days [8,9]. Farmers traditionally use antibiotics in an attempt to control bacterial diseases in aquaculture. However, the effectiveness of antibiotics is rapidly decreasing as a result of the emergence and spread of (multiple) antibiotic resistance [10,11]. Another unfortunate consequence is that aquaculture is one of the major sources of antibiotic resistance genes in human pathogens [12]. Hence, for the sustainable development of the aquaculture industry, there is an urgent need for novel strategies to control bacterial infections [13].

A promising novel approach to control bacterial diseases, is antivirulence therapy, which aims at disarming bacterial pathogens rather than killing them or inhibiting their growth [14]. In order to achieve this, antivirulence therapy focuses on the inhibition of virulence factors, i.e., compounds and cell structures synthesized by bacterial pathogens that enable them to infect their host [15,16]. This definition comprises secreted products such as toxins, siderophores, lytic enzymes, and exopolysaccharides, as well as cell-associated structures such as secretion systems, adhesins, and flagella [17,18]. Antivirulence therapy presents a promising alternative to traditional antibiotics in combating bacterial diseases as it can prevents pathogens from attacking their host [15]. Ideal virulence inhibitors target only the virulence factors of pathogenic bacteria without affecting their viability. As a result, the selective pressure on pathogens to become resistant is relatively mild, which delays or even prevents the spread of resistance, as we have recently shown specifically for the three-channel quorum sensing system of *V. campbellii* [19]. An additional advantage of this approach is that it does not kill or inhibit the growth of other microbes, particularly those with beneficial effects on the host [17].

The virulence regulatory mechanism that has been studied most intensively in the framework of antivirulence therapy, is quorum sensing, bacterial cell-to-cell communication [15]. Vibrios belonging to the *Harveyi* clade contain a three-channel quorum sensing system that controls the production of the master regulator LuxR, which in turn controls transcription of virulence factors (including the *vhp* metalloprotease gene and flagellar genes) and which is essential for full virulence of these bacteria [4]. Reference [20] previously reported a small molecule, 1-(5-bromothiophene-2-sulfonyl)-1H-pyrazole, named Qstatin, as a quorum sensing inhibitor in *Vibrio vulnificus*. They documented that Qstatin inhibited the function of SmcR, the LuxR homologue of *V. vulnificus*. Qstatin interacts with SmcR, thereby altering its DNA-binding properties and thus preventing the expression of quorum sensing-regulated virulence factors. Qstatin decreased *V. vulnificus* chemotactic motility, protease expression, biofilm dispersion, and virulence to brine shrimp (*Artemia franciscana*). Most importantly, Qstatin had no effect on the growth of *V. vulnificus* [20]. Since the three-channel quorum sensing system is required for full virulence of *Harveyi* clade vibrios, we reasoned that Qstatin might be an interesting virulence inhibitor to control disease caused by these bacteria as well.

In order to determine the impact of Qstatin on the virulence of shrimp-pathogenic vibrios belonging to the *Harveyi* clade in this study, we first used the quorum sensing model strain *V. campbellii* BB120 to investigate the effects of Qstatin on growth kinetics, quorum sensing-regulated bioluminescence, swimming motility, biofilm formation and proteolytic activity, as well as on the expression of the genes related with these activities. After establishing the dose–response relationship, we utilized 12 other shrimp-pathogenic strains belonging to the species *V. parahaemolyticus*, *V. harveyi* and *V. campbellii*, to explore the protective efficacy of Qstatin, using a highly controlled model system with gnotobiotic brine shrimp (*Artemia franciscana*) larvae.

## 2. Materials and Methods

### 2.1. Bacterial Strains, Culture Media, and Reagents

**Figure A1:**
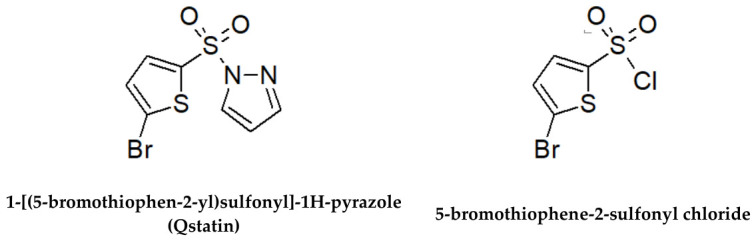
Compounds used in this study.

All strains used in this study are listed in Table 1. All *Vibrio* strains were grown in Luria–Bertani medium (LB) containing 35 g/L NaCl (LB_35_) at 28 °C under 100 min^−1^ constant agitation. Qstatin (Figure A1) was purchased from BIO-Connect (Huissen, The Netherlands), dissolved in DMSO at concentrations of 0.2, 2, 20, 50, 100, and 200 mM, and stored at −80 °C until use. In addition, 5-bromothiophene-2-sulfonyl chloride was purchased from Merck (Darmstadt, Germany), dissolved in DMSO at concentrations of 0.1, 1, 10, 50, and 100 mM, and stored at −80 °C until use. In all assays, all treatments received the same volume of DMSO.

### 2.2. Bacterial Growth and Bioluminescence Assays

Growth and bioluminescence assays were performed as described previously [29] with some modifications. After overnight growth, *V. campbellii* BB120 was diluted to an OD_600_ of 0.1, inoculated into fresh LB_35_ broth and continued to grow for 4 h to the mid-exponential phase and diluted again to an OD_600_ of 0.1. Qstatin was added to the suspensions at final concentrations of 0, 0.2, 2, 20, 50, 100, and 200 µM. Then, 5-bromothiophene-2-sulfonyl chloride was added to the suspensions at final concentrations of 0.1, 1, 10, 50, and 100 µM. After mixing, 200 µL volumes of these mixtures were transferred to the wells of a polystyrene 96-well plate and cultured at 28 °C for 12 h. The optical density at 600 nm (OD_600_) and bioluminescence of each culture were measured every hour using a Tecan Infinite M200Pro plate reader (Tecan, Mechelen, Belgium). Growth and bioluminescence curves were determined for three independent cultures. To verify that Qstatin specifically affects LuxR-regulated bioluminescence, *E. coli* DH5alpha pAKlux1 was included as a control strain. This strain lacks the LuxR-dependent quorum sensing system, and contains the luminescence genes under control of a constitutive promoter [30].

### 2.3. Impact of Qstatin on Viability in a Nutrient-Limited Environment

After overnight growth, *V. campbellii* BB120 was inoculated into fresh LB_35_ at OD_600_ = 0.1 and cultured for 4 h to the mid-exponential phase. LB_35_ medium was 1000-fold diluted in a 35 g/L NaCl solution. *Vibrio campbellii* BB120 (OD_600_ = 1) was diluted 1000-fold in this solution. Qstatin was subsequently added at 0, 0.2, 2, 20, 50, 100, and 200 µM to the diluted bacterial suspensions. After 1 h incubation at 28 °C, all suspensions were plated onto LB_35_ agar. Colonies were counted after 24 h. All treatments were performed in triplicate.

### 2.4. Proteolytic Activity Assay

Proteolytic activity was assessed according to [31] with some modifications. Double-strength LB_35_ (40 g/L agar) was mixed with an equal volume of 4% skim milk powder suspension (Oxid, Basingstoke, Hampshire, UK) sterilized separately at 121 °C for 5 min. After cooling to approximately 50 °C, Qstatin was added at final concentrations of 0.2, 2, 20, 50, 100, and 200 µM, mixed thoroughly, and then poured into Petri plates to prepare protease assay plates. *V. campbellii* BB120 was grown overnight and diluted to an OD_600_ of 1.0 with fresh LB_35_ broth. In total, 3 µL aliquots were spotted onto the middle of the assay plates. All assays were done in triplicate at a minimum. The diameters of clearing zones and bacterial colonies were measured after 48 h of upright incubation.

### 2.5. Swimming Motility Assay

Swimming motility assays were performed as described previously [32]. Qstatin with final concentrations of 0, 0.2, 2, 20, 50, 100, and 200 µM was added to autoclaved LB_35_ soft agar (LB_35_ containing 0.2% agar), mixed thoroughly, and then poured into Petri plates before the agar solidified. Three microlitre aliquots of freshly grown *V. campbellii* BB120 (OD_600_ = 1.0) were inoculated onto the centre of the plates. Plates were incubated upright at 28 °C for 18 h, after which the diameters of the motility zones were measured for each plate. All assays were done with freshly prepared media in six replicates per treatment.

### 2.6. Biofilm Formation Assay

Biofilm formation was quantified by crystal violet staining, as described previously [33]. An overnight culture of *V. campbellii* BB120 was diluted in fresh LB_35_ broth to an OD_600_ of 0.1 and supplemented with Qstatin (final concentrations: 0, 2, 20, 50, 100, and 200 µM). After vortex mixing, 200 µL aliquots were dispensed into the wells of a sterile 96-well polystyrene plate, which were then incubated statically at 28 °C for 48 h. Following incubation, media were gently aspirated and wells were rinsed with 300 µL PBS three times to remove non-adherent cells. After air-drying, 200 µL methanol was added to each well and the plate was incubated at room temperature for 20 min to fixate adherent bacteria. The methanol was subsequently removed, and the plate was inverted overnight at room temperature until it was completely dry. Fixated biofilms were stained with 200 µL of a 0.1% crystal violet solution for 15 min at room temperature. The stain was then removed, and the plates were rinsed extensively with distilled water until no residual stain was observed in the effluent. After complete air-drying, bound crystal violet was dissolved in 200 µL of 95% ethanol per well for 30 min, and absorbance was measured at 570 nm with a Tecan Infinite M200Pro plate reader (Tecan, Mechelen, Belgium). Sterile LB_35_ broth served as the negative control, and all reported optical density values were blank corrected by subtracting control well readings.

### 2.7. Axenic Hatching and Rearing of Brine Shrimp Larvae

In total, 200 milligrams of high-quality hatching cysts of *Artemia franciscana* (EG^®^ Type; INVE Aquaculture, Baasrode, Belgium) were hydrated in 18 mL of tap water for 1 h. The process for obtaining sterile cysts and larvae were adapted from methods described previously by [34,35]. In brief, 660 µL of NaOH (32%) and 10 mL NaOCl (50%) were added to the hydrated cyst suspension to facilitate decapsulation. The decapsulation was stopped after 2 min by adding 14 mL of Na_2_S_2_O_3_ (10 g/L). The decapsulated cysts were washed with filtered and autoclaved artificial seawater (containing 35 g/L of instant ocean synthetic sea salt; Aquarium Systems, Sarrebourg, France). The cysts were resuspended in a 50 mL falcon tube containing 30 mL of filtered and autoclaved artificial seawater, and then hatched for 28 h on a rotor (four rotations per minute) at 28 °C with aeration and constant illumination (2000 lux). After hatching, 1 mL of rearing water was inoculated into 9 mL of LB_35_ broth and incubated at 28 °C for 24 h to verify cyst sterility. After almost all *Artemia* had hatched, groups of 20 larvae were transferred to sterile 15 mL tubes containing 10 mL of filtered and autoclaved artificial seawater. The nauplii were fed with an autoclaved LVS3 bacterial suspension [28] (at 10^7^ CFU/mL) and the pathogenic vibrios were added subsequently. Finally, all tubes were put on a rotor (four rotations per minute) at 28 °C with constant illumination (2000 lux).

### 2.8. Brine Shrimp Challenge Tests

The impact of Qstatin on the virulence of vibrios was determined using a standardized challenge test with gnotobiotic of brine shrimp larvae. Challenge tests were performed as described by [34]. Briefly, tested strains of *Vibrio* and Qstatin were added into the brine shrimp rearing water at the start of the experiment. The inoculum concentration of the vibrios was 10^6^ CFU/mL, and the final concentrations of Qstatin were 0, 0.2, 2, 20, 50, 100, and 200 µM. The survival of *Artemia* was counted 48 h after the addition of the vibrios. Each treatment was carried out in triplicate, and each experiment was repeated twice using a different batch of larvae to verify the reproducibility. After each challenge test, 1 mL rearing water of the negative control (no live bacteria added) was inoculated into 9 mL of fresh LB_35_ broth and incubated at 28 °C for 48 h to check the sterility of the larvae. In addition, the quantity of vibrios in the brine shrimp rearing water after 48 h was measured by plate counting on LB_35_ agar.

### 2.9. RNA Extraction and Reverse Transcription

Overnight cultures of *V. campbellii* strains BB120 and S01 were diluted to an OD600 of 0.1 in fresh LB_35_ medium and incubated for 6 h with or without 100 µM Qstatin until the late exponential phase (OD_600_ ≈ 1.0). The cultures were centrifuged at 4 °C, 5000× *g*, for 10 min, after which the supernatants were discarded, and the pellets were retained for RNA extraction. For the specific analysis of the lateral flagellar genes *lafA* and *lafK*, an alternative sampling method was employed: 5 µL of a bacterial suspension adjusted to OD_600_ = 1.0 was spotted onto LB_35_ agar containing 0.75% agar, supplemented with or without 100 µM Qstatin, and incubated at 28 °C. After incubation for 48 h, bacteria were collected from the plates by rinsing with sterile PBS, centrifuged under the same conditions, and the pellets were retained for RNA extraction.

RNA extraction was performed as described previously [32,36] using the SV Total RNA Isolation System (Promega, Leiden, The Netherlands). The quantity of extracted RNA was measured spectrophotometrically (NanoDrop Technologies, Wilmington, DE, USA). The RNA quality was confirmed with agarose gel electrophoresis, and the RNA samples were stored at −80 °C.

Reverse transcription was carried out with the RevertAid H Minus First Strand cDNA Synthesis Kit (Thermo Fisher Scientific, Waltham, MA, USA) in accordance with the manufacturer’ s instructions. Briefly, a mixture of 1 µg RNA, 2 µL random primer solution, and nuclease-free H_2_O was mixed to a total volume of 12 µL. Then, 4 µL of 5× reaction buffer, 1 µL of RiboLock RNase inhibitor (20 U/µL), 2 µL of 10 mM dNTP Mix, and 1 µL of RevertAid H Minus M-MuLV reverse transcriptase (200 U/µL) were added. The 20 µL cDNA synthesis reactions were incubated at 25 °C for 5 min, followed by 60 min at 42 °C. The reaction was terminated by heating at 70 °C for 5 min and then cooled to 4 °C. Finally, cDNA samples were stored at −20 °C for further use.

### 2.10. Quantitative Reverse Transcriptase Real-Time PCR (RT-qPCR)

RT-qPCR was used to quantify the expression level of all tested genes and was performed in a QuantStudio^TM^ Real-Time PCR Systems (Thermo Fisher Scientific, USA) by using the Maxima SYBR Green/ROX qPCR Master Mix (2×) (Thermo Fisher Scientific, USA) as descried previously [32,36]. The total reaction volume of 25 µL was prepared following the manufacturer’s instructions by mixing 12.5 µL Maxima SYBR Green Master Mix, 0.3 µM each of forward and reverse primers (Table 2), 1 µL of template cDNA, and nuclease-free water.

The PCR amplification protocol comprised an initial denaturation at 95 °C for 10 min, followed by 40 cycles per reaction, with each cycle consisting of denaturation at 95 °C for 15 s, annealing at 60 °C for 30 s, and extension at 72 °C for 30 s. Each treatment was performed on three biological replicates, each with two technical replicates. The RNA polymerase A subunit (*rpoA*) was used as an endogenous control. The comparative threshold cycle method (2^−ΔΔCT^) was used to analyze the relative mRNA levels of the tested genes [37].

**Table 2:** Primers used in this study for RT-qPCR.

Gene	Primer Sequence (5′–3′)	Product Size (bp)	References
*flaA*	F: CTGCGGGTCTTCAAATCTC	205	[32]
	F: CTGCGGGTCTTCAAATCTC		
*flaK*	F: ATTGCCCGTTGATGATTTG	128	[32]
	R: CTTCTGTGCCCGATACTTGT		
*lafA*	F: TAACTTCGCATCGCTTGTAAC	210	[32]
	R: TCGTCTGCTGCTGAGTTGATA		
*lafK*	F: GAGCCAAATGAACACCTCG	111	[32]
	R: AACAATCGCAATCACCACA		
*vpsR*	F: GCAGTTCTGATGTCTGATAGCG	132	[38]
	R: CTCCAACACCACCAGCAATG		
*vpsT*	F: TTACGGCTAAACCACATA	127	[38]
	R: CGATTACAACGGAAGAGT		
*vhp*	F: CTGAACGACGCCCATTATTT	201	[39]
	R: CGCTGACACATCAAGGCTAA		
*pirA*	F: TTGGACTGTCGAACCAAACG	135	[40]
	R: GCACCCCATTGGTATTGAATG		
*pirB*	F: ACTAGGCAAGGCTCATAAATATGACG	102	[41]
	R: ATTGCTTCAGGTCCATTGGCAATAA		
*rpoA*	F: TGGTCGTGGTTATGTTCC	189	This study
	R: AGAGTACCGTTCGTTTCC		

### 2.11. Statistics

Statistical analyses were performed using SPSS software (version 29, IBM, Brussels, Belgium). After verifying that conditions of normality and homogeneity of variances were met, one-way ANOVA followed by a Duncan’s post hoc test or independent samples *t*-test were used to compare untreated versus Qstatin-treated groups and to evaluate differences among treatments with varying Qstatin concentrations, with statistical significance set at *p* < 0.01.

## 3. Results

### 3.1. Impact of Qstatin on Growth Kinetics and Quorum Sensing-Regulated Bioluminescence of V. campbellii

**Figure 1:**
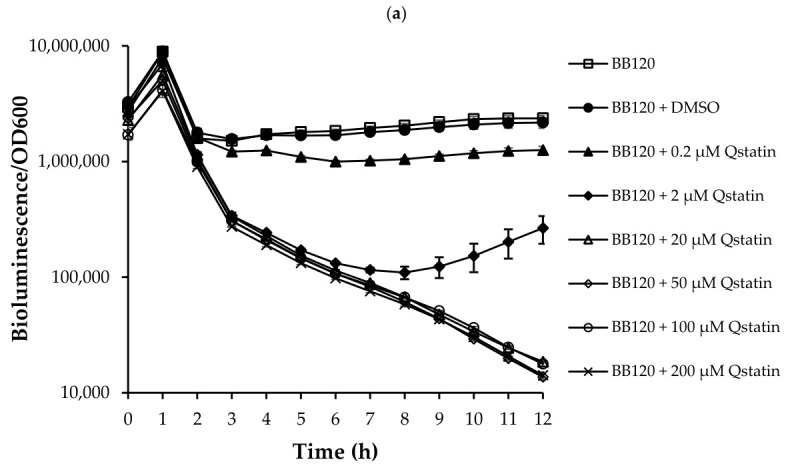

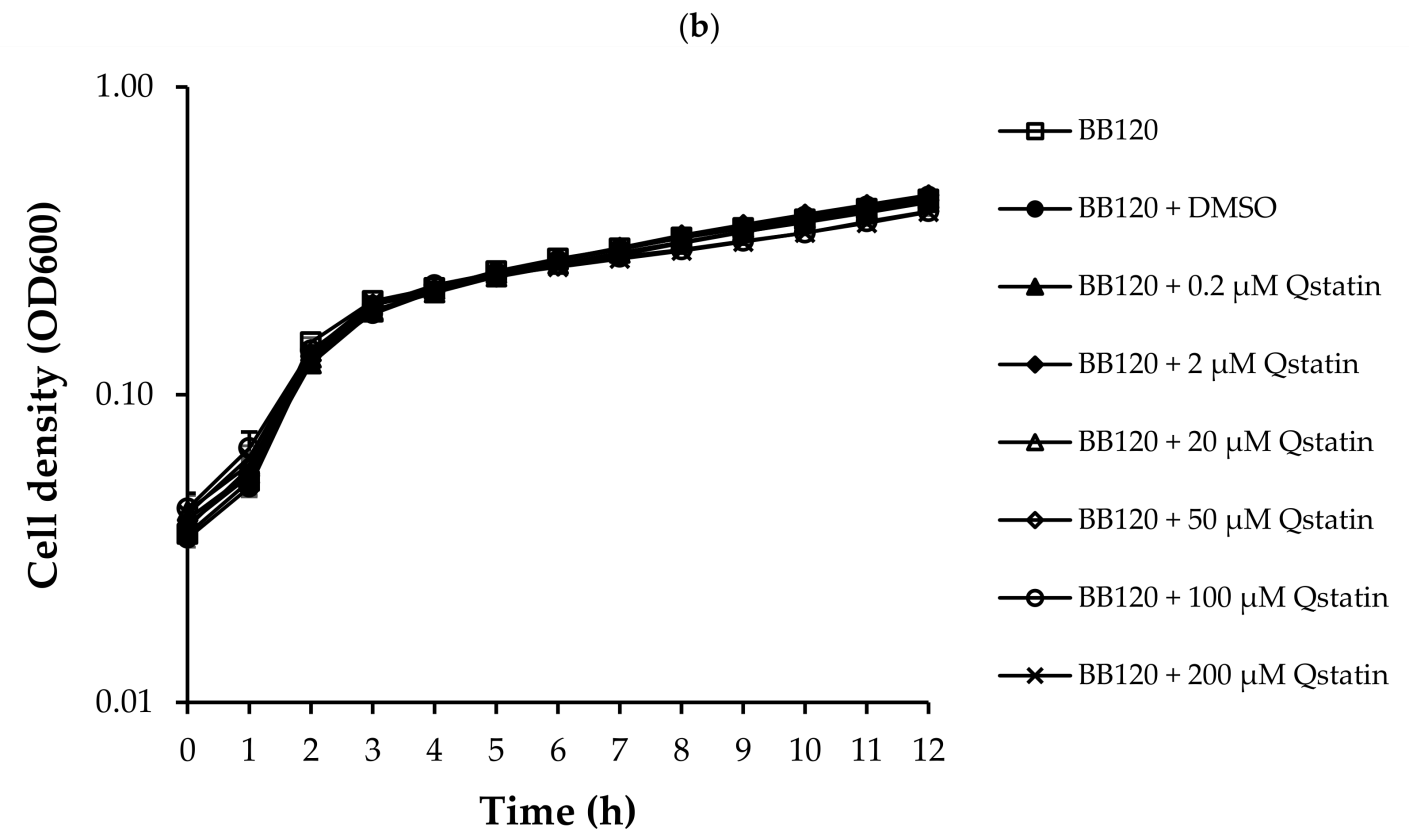
Quorum sensing-regulated bioluminescence (**a**) and growth kinetics (**b**) of *V. campbellii* BB120 in LB_35_ medium with and without Qstatin (at 0.2, 2, 20, 50, 100, and 200 µM). Error bars represent standard deviations of three replicate cultures.

**Figure A2:**
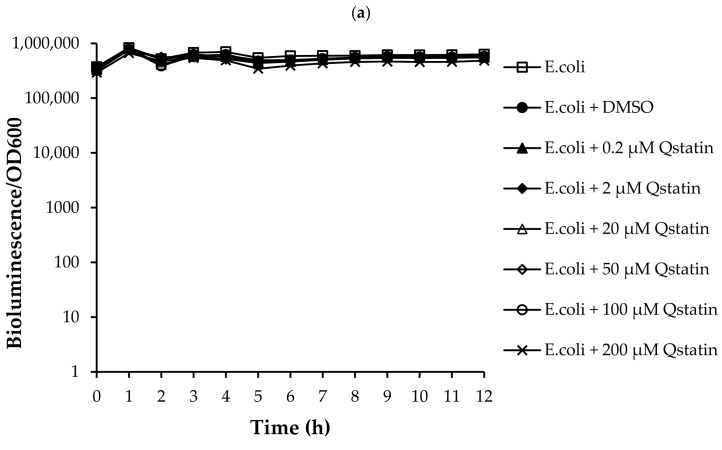

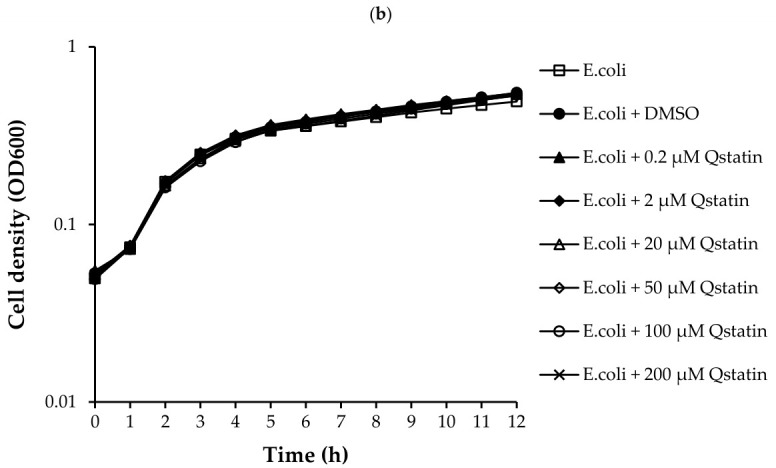
Quorum sensing-independent bioluminescence (**a**) and growth kinetics (**b**) of *E. coli* DH5alpha pAKlux1 in LB medium with and without Qstatin (0.2, 2, 20, 50, 100 and 200 μM). Error bars show the standard deviations of three replicate culture.

Given that bioluminescence in *V. campbellii* is controlled by the three-channel quorum sensing system [42], we used bioluminescence intensity to confirm that Qstatin inhibited the quorum sensing system. As shown in Figure 1a, Qstatin significantly decreased the bioluminescence of *V. campbellii* BB120 at concentrations of 0.2 µM and above. Treatment with 2 µM Qstatin caused a maximal inhibition of 95% after 8 h, followed by a gradual attenuation. At higher concentrations, more than 99% inhibition of bioluminescence was observed and this lasted until the end of the experiment. Importantly, Qstatin had no significant impact on the growth kinetics of *V. campbellii* at any of the tested concentrations (Figure 1b), nor did it affect constitutive bioluminescence in the control strain *E. coli* DH5alpha pAKlux1 (Figure A2), confirming that the inhibition of bioluminescence in *V. campbellii* was specifically caused by quorum sensing inhibition.

**Figure A3:**
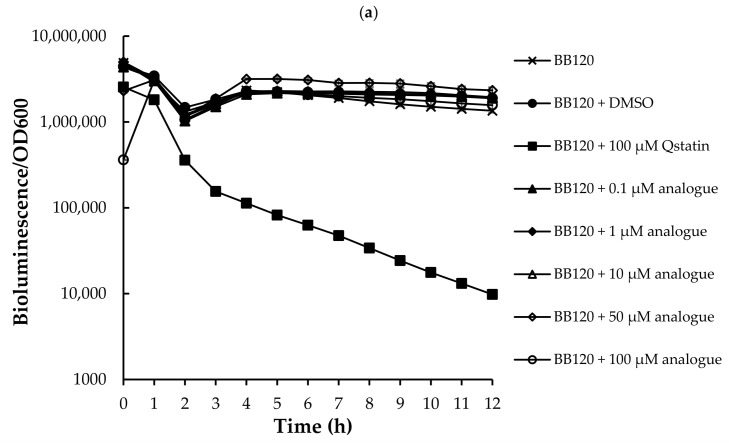

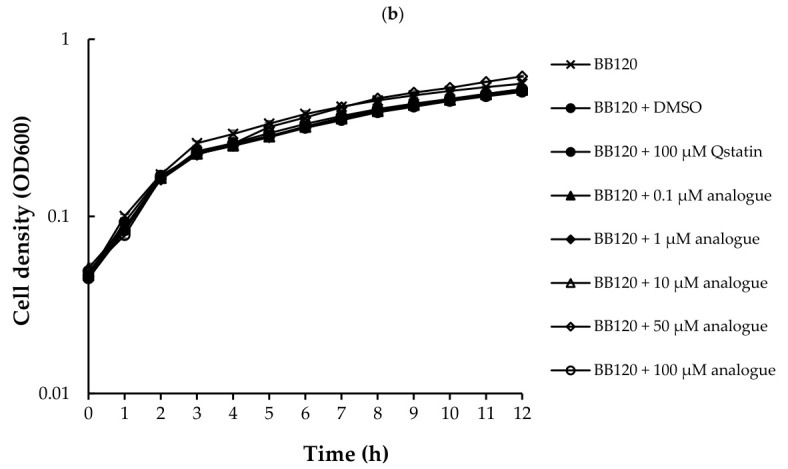
Quorum sensing-controlled bioluminescence (**a**) and growth kinetics (**b**) of *V. campbellii* BB120 in LB_35_ medium with and without the Qstatin analogue 5-bromothiophene-2-sulfonyl chloride at different concentrations. Qstatin at 100 µM was used as a control. Error bars show the standard deviations of three replicate cultures.

Based on previous work with thiophenones [43], we hypothesized that the bromothiophene moiety of Qstatin might be responsible for its quorum sensing-disrupting activity. Consequently, we determined the impact of the Qstatin analogue 5-bromothiophene-2-sulfonyl chloride (Figure A1) on quorum sensing-regulated bioluminescence of *V. campbellii*. However, the compound had no impact on bioluminescence of the strain (Figure A3).

### 3.2. Impact of Qstatin on the Survival of V. campbellii in a Nutrient-Limited Environment

**Figure 2:**
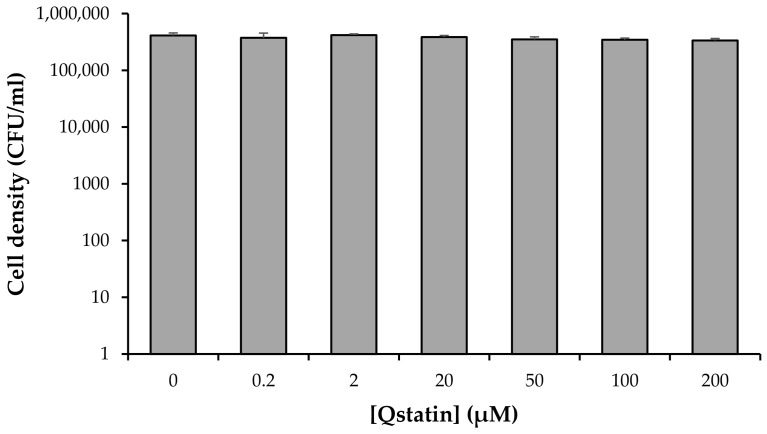
Impact of Qstatin on survival of *V. campbellii* BB120 in nutrient-poor conditions (i.e., 1000-fold diluted LB_35_ medium), as determined by plate counting. Error bars represent standard deviations of three replicate cultures.

In nutrient-rich environments, subtle compound toxicity may be obscured by the optimal conditions, whereas it can be revealed when the bacteria are incubated in more harsh, nutrient-limited conditions, as we reported previously for pyrogallol [44]. To exclude that Qstatin exhibits such subtle toxicity, Qstatin was applied in a nutrient-limited environment (i.e., 1000-fold diluted LB_35_ medium), and the survival of *V. campbellii* after 1 h incubation was determined by plate counting. There were no significant differences in cell counts between Qstatin-treated groups and untreated controls (Figure 2).

### 3.3. Impact of Qstatin on the Expression of Quorum Sensing-Regulated Genes in V. campbellii

To further substantiate transcriptional inhibition of quorum sensing-regulated virulence genes by Qstatin, we analyzed the expression of the polar flagellum master regulator and flagellin genes *flaK* and *flaA*, the lateral flagella regulator and flagellin genes *lafK* and *lafA*, the *Vibrio* polysaccharide (VPS) synthesis regulator genes *vpsR* and *vpsT*, and the *vhp* metalloprotease gene. In addition to this, we also assessed the impact of Qstatin on the mRNA levels of the AHPND toxin genes *pirA* and *pirB* in AHPND-causing strain S01. The results demonstrated that treatment with 100 µM Qstatin significantly inhibited the expression of most genes, though the extent of inhibition varied (Table 3).

In both BB120 and S01, Qstatin markedly downregulated genes involved in polar flagellar synthesis. The expression of the polar flagellin gene *flaA* was reduced 1.7- to 2.0-fold, while the polar flagellar master regulator gene *flaK* was more profoundly inhibited, with mRNA levels undetectable after Qstatin treatment. In contrast, genes encoding lateral flagella (*lafA* and *lafK*) were not significantly affected. Qstatin also exerted strong inhibitory effects on biofilm matrix regulators. The expression of *vpsR*, the main activator of *Vibrio* polysaccharide production, was reduced by 3.3- to 5.0-fold. Similarly, *vpsT*, a secondary biofilm regulator, was markedly inhibited, with expression decreased about 10-fold in both strains. Further, the expression of the *vhp* metalloprotease gene was consistently inhibited by approximately 2.5-fold in both BB120 and S01. Finally, Qstatin did not significantly affect the mRNA levels of the AHPND toxin genes *pirA* and *pirB* in strain S01.

### 3.4. Impact of Qstatin on Swimming Motility, Biofilm Formation, and Proteolytic Activity of V. campbellii

**Figure 3:**
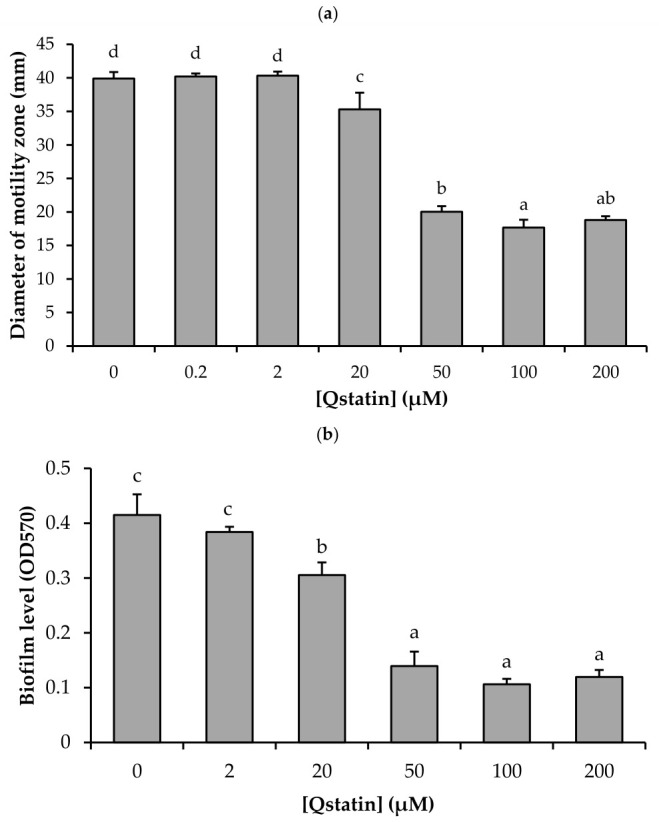

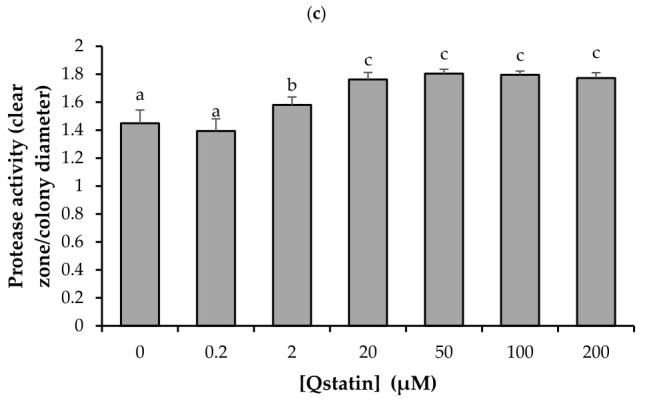
Impact of Qstatin on (**a**) swimming motility of *V. campbellii* BB120 on soft agar plates, (**b**) biofilm levels on polystyrene 96-well plates, and (**c**) proteolytic activity on casein-containing agar. Error bars represent standard deviations of six (motility) and three (other assays) replicate cultures. Different letters indicate significant differences (one-way ANOVA with Duncan’s post hoc test; *p* < 0.01).

We further investigated the impact of Qstatin on the three quorum sensing-regulated virulence factors related to the genes that were differentially expressed: swimming motility, biofilm formation, and proteolytic activity. Qstatin significantly decreased the swimming motility of *V. campbellii* BB120 in a concentration-dependent manner (Figure 3a). Quantification of motility zone diameters showed a maximal decrease of 57% at 100 µM.

Crystal violet staining revealed that Qstatin also significantly decreased biofilm formation of *V. campbellii* BB120 in a concentration-dependent manner (Figure 3b). Quantification of biofilm-bound crystal violet revealed a maximal decrease of 76% at 100 µM.

Unexpectedly, Qstatin did not decrease but rather slightly increased protease activity of *V. campbellii* (Figure 3c). The clearing zone/colony diameter ratio showed an increase of 24% at 100 µM. However, such a small effect is probably not biologically relevant.

### 3.5. Impact of Qstatin on the Virulence of Harveyi Clade Vibrios in the Gnotobiotic Brine Shrimp (Artemia franciscana) Model

**Figure 4:**
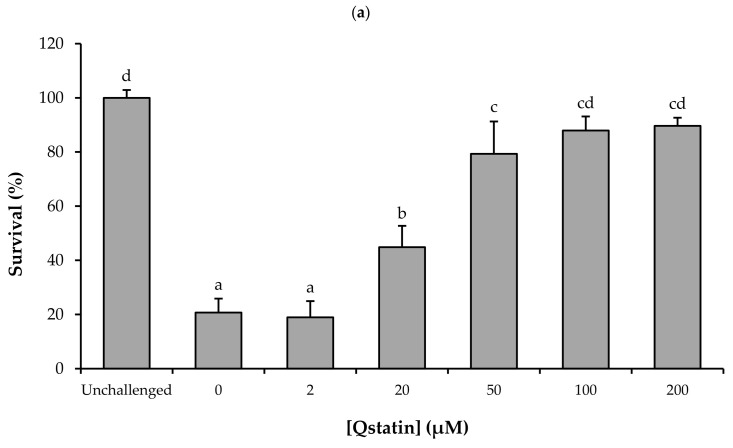

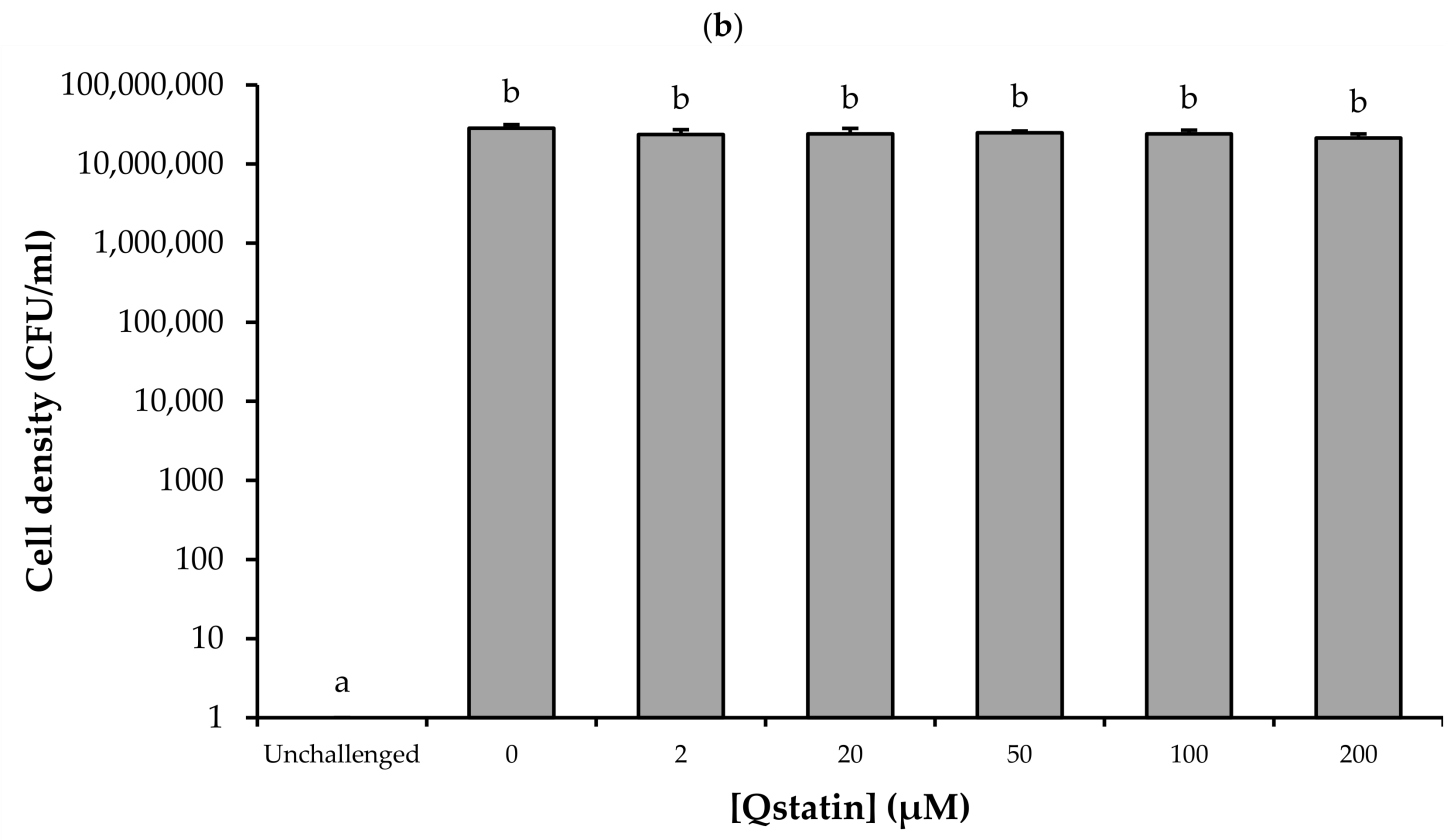
(**a**) Survival of gnotobiotic brine shrimp larvae after 48 h of challenge with *V. campbellii* BB120 and treated with different concentrations of Qstatin. (**b**) Cell density of *V. campbellii* BB120 in brine shrimp rearing water after 48 h of challenge. Error bars represent standard deviations of three shrimp cultures. Different letters indicate significant differences (one-way ANOVA with Duncan’s post hoc test; *p* < 0.01). “Unchallenged” indicates brine shrimp without addition of *V. campbellii* that were otherwise treated in the same way as challenged brine shrimp.

To evaluate the protective efficacy of Qstatin against pathogenic *Harveyi* clade vibrios, we used the highly controlled gnotobiotic brine shrimp assay. In order to determine the optimal dose, we first performed a challenge test with model strain *V. campbellii* BB120 and treated the challenged brine shrimp larvae with different doses of Qstatin. Qstatin significantly increased the survival of challenged brine shrimp when added to the rearing water at 20 µM or more, with complete protection (no significant difference in survival when compared to unchallenged larvae) at 100 µM or more (Figure 4a). No significant difference in bacterial cell density in the brine shrimp rearing water was observed among the different infected groups (Figure 4b).

**Figure 5:**
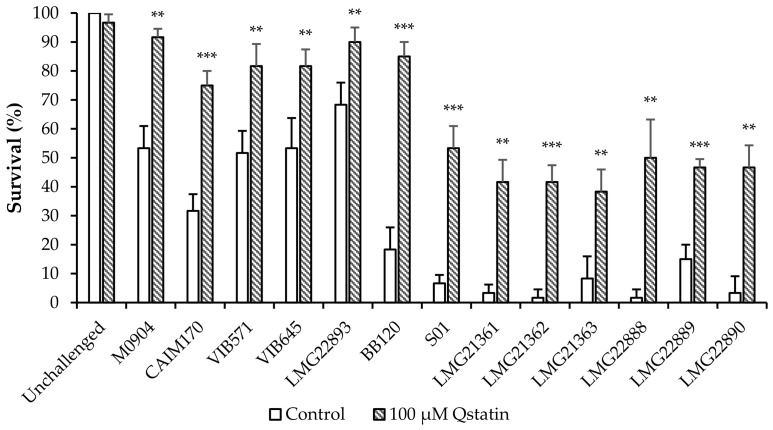
Survival of gnotobiotic brine shrimp larvae with and without 100 µM Qstatin after 48 h of challenge with different shrimp-pathogenic *Harveyi* clade *Vibrio* strains belonging to the species *V. parahaemolyticus* (M0904 and CAIM170), *V. harveyi* (VIB571, VIB645 and LMG 22893), and *V. campbellii* (BB120, S01, LMG 21361, LMG 21362, LMG 21363, LMG 22888, LMG 22889 and LMG 22890). The error bars represent the standard deviations of three shrimp cultures. Asterisks indicate significant differences when compared with the treatment with the same strain, but without Qstatin (Independent samples *t*-test; ** *p* < 0.01, *** *p* < 0.001). “Unchallenged” indicates brine shrimp without addition of *V. campbellii* that were otherwise treated in the same way as challenged brine shrimp.

Based on these results, we decided to retain the 100 µM treatment for a subsequent challenge tests with twelve other *Harveyi* clade strains (belonging to the species *V. campbellii, V. harveyi* and *V. parahaemolyticus*). The results demonstrated that treatment with Qstatin significantly increased the survival of brine shrimp larvae for all tested strains (Figure 5).

## 4. Discussion

The increasing challenge of antibiotic resistance in aquaculture underscores the urgent need for novel strategies to control bacterial diseases. Antivirulence therapy, which aims at disarming pathogens without affecting their viability, represents a promising alternative by minimizing selective pressure for resistance [15]. In this study, we demonstrated that Qstatin, a known quorum sensing inhibitor in *V. vulnificus* [20], significantly decreased the virulence of pathogenic vibrios belonging to the *Harveyi* clade by specifically interfering with their three-channel quorum sensing system. This resulted in the protection of brine shrimp from different shrimp-pathogenic strains belonging to the species *V. campbellii, V. harveyi*, and *V. parahaemolyticus*, including AHPND-causing strains.

The specific inhibition of quorum sensing-regulated bioluminescence in *V. campbellii* BB120 (Figure 1a), without affecting viability (even under nutrient-limited conditions), confirms the antivirulence character of Qstatin. This is consistent with its previously described mode of action of altering the DNA-binding properties of the “high density” master regulator of the three-channel quorum sensing system of vibrios [20]. Control experiments using an engineered *E. coli* strain, in which bioluminescence is independent of quorum sensing [43], confirmed that the observed effect on bioluminescence is specifically due to quorum sensing inhibition and not to general metabolic defects, cytotoxicity, or inhibition of bioluminescence biochemistry. This specificity and lack of antibacterial activity is particularly valuable for applications, as it will likely preserve the beneficial microbiota while targeting the pathogenicity of the vibrios [17]. Collectively, these results underscore Qstatin as a promising quorum sensing-specific inhibitor. We performed a small structure–activity analysis to determine whether the 5-bromothiophene-2-sulfonyl moiety of Qstatin was responsible for its quorum sensing-inhibiting activity. However, 5-bromothiophene-2-sulfonyl chloride did not affect the bioluminescence of *V. campbellii*, suggesting that the pyrazole moiety is required for the activity of Qstatin.

In order to investigate the antivirulence effect of Qstatin, we evaluated its effect on the expression and function of several key virulence determinants in *V. campbellii*. We observed a significant downregulation of the expression of quorum sensing-regulated virulence genes and the concomitant attenuation of the corresponding phenotypes, confirming that Qstatin suppresses virulence by targeting quorum sensing-regulated gene expression. Indeed, the complete inhibition of the expression of *flaK* (Table 3), which encodes the polar flagellar master regulator, corresponds with the significant reduction in swimming motility we observed (Figure 3a). This indicates that Qstatin disrupts the regulatory hierarchy controlling flagellar assembly and function, essential for host colonization [32,45]. Similarly, the dramatic downregulation of *vpsR* and *vpsT* (3.3- to 10-fold reduction) provides a mechanistic explanation for the observed decrease in biofilm formation (Figure 3b), as these genes encode master regulators of *Vibrio* polysaccharide (VPS) production [38]. The *vps* genes regulate the production of exopolysaccharides, which form the biofilm matrix [46]. In addition to this, the *vhp* metalloprotease gene was also downregulated. This finding is consistent with previous reports establishing *vhp* metalloprotease as a quorum sensing-controlled virulence gene in *Harveyi* clade vibrios [39,47]. The decrease in the expression of the *vhp* gene was, however, not reflected in a decreased protease activity on casein agar. This might be due to the fact that *Harveyi* clade vibrios contain multiple protease genes, and not all of them are controlled by the three-channel quorum sensing system (e.g., the serine protease gene *srp* is unaffected by the three-channel quorum sensing system [48]). Therefore, the decrease in expression of one of the proteases might have no impact on overall protease activity as observed in this study. Notably, Qstatin did not alter the expression of the plasmid-encoded *pirAB* toxin genes in the AHPND-causing S01 strain. This indicates that these genes are not controlled by the “high density” master regulator of the three-channel quorum sensing system.

The translational relevance of these molecular effects was demonstrated in standardized gnotobiotic brine shrimp challenge assays. At 100 μM, Qstatin significantly increased survival of brine shrimp challenged with each of the 13 tested *Harveyi* clade strains (Figure 4 and Figure 5). Comparable bacterial densities in the rearing water across all test groups confirmed that the observed protection resulted from virulence inhibition rather than antibacterial activity (Figure 4). This ability to reduce pathogen virulence without affecting bacterial viability aligns Qstatin with the antivirulence concept and supports the ecological advantage of antivirulence therapy [15,19]. Although we did not directly demonstrate quorum sensing inhibition in all 13 strains, given the conservation of the three-channel quorum sensing system within the *Harveyi* clade, we hypothesize that Qstatin has the same mode of action in all strains. Notably, Qstatin also attenuated the virulence of strains that cause AHPND. Although Qstatin did not affect the expression of the plasmid-encoded *pirAB* toxins, the inhibition of other key virulence determinants (e.g., motility and biofilm formation) apparently was sufficient to confer protection to the host.

## 5. Conclusions

This study provides a comprehensive analysis of Qstatin’s antivirulence activity against shrimp-pathogenic *Harveyi* clade vibrios. By demonstrating concurrent inhibition of virulence factors at transcriptional and phenotypic levels, and by validating the protective efficacy in a highly controlled in vivo model system, we establish a robust foundation for its potential application. Qstatin thus represents a promising candidate for sustainable disease management in (shrimp) aquaculture, given the fact that *Harveyi* clade vibrios are major bacterial pathogens of aquatic organisms. Future research could investigate cheaper analogues of Qstatin, optimizing the mode of administration for aquaculture (feed application will probably be more efficient), and investigate potential synergies with other virulence inhibitors. The selective pressure dynamics during long-term exposure also merit investigation to fully assess the risk of resistance development.

## Figures and Tables

**Table 1:** Bacterial strains used in this study.

Strain	Relevant Features	References
*Vibrio parahaemolyticus*		
M0904	AHPND-causing strain; isolated from the hepatopancreas of diseased shrimp (*Penaeus vannamei*), Mexico.	[21]
CAIM 170	Isolated from the hemolymph of diseased shrimp (*Penaeus* spp.), Mexico.	[22]
*Vibrio harveyi*		
VIB 571	Isolated from sea bass (*Dicentrarchus labrax*), Spain.	[23]
VIB 645	Isolated from sea bass (*Dicentrarchus labrax*), Tunisia.	[23]
LMG 22893	Isolated from the hemolymph of diseased shrimp (*Penaeus* spp.), Mexico.	[22]
*Vibrio campbellii*		
BB120	Model strain for studying quorum sensing in vibrios; previously classified as *V. harveyi.*	[24]
S01	AHPND-causing strain; isolated from a shrimp (*Penaeus* spp.) farm, China.	[25]
LMG 21361	Isolated from seawater from shrimp (*Litopenaeus* spp.) broodstock tanks, Mexico.	[26,27]
LMG 21362	Isolated from seawater from shrimp (*Litopenaeus* spp.) broodstock tanks, Mexico.	[26,27]
LMG 21363	Isolated from lymphoid organs from diseased juveniles (*Penaeus* spp.) Philippines.	[27]
LMG 22888	Isolated from seawater from shrimp (*Litopenaeus* spp.) broodstock tanks, Mexico.	[26,27]
LMG 22889	Isolated from seawater from shrimp (*Litopenaeus* spp.) broodstock tanks, Mexico.	[26,27]
LMG 22890	Isolated from seawater from shrimp (*Litopenaeus* spp.) broodstock tanks, Ecuador.	[26,27]
*Aeromonas* sp.		
LVS3	Feed for brine shrimp larvae.	[28]

**Table 3:** Relative mRNA levels of virulence genes during incubation of *V. campbellii* strains BB120 and S01 in LB_35_ broth in the absence and presence of 100 µM Qstatin (average ± standard deviation of three independent cultures).

		Relative Expression ^1^			
		BB120		S01	
Gene	Function	Untreated	+Qstatin	Untreated	+Qstatin
*flaA*	Polar flagellar flagellin	1.0 ± 0.3	0.6 ± 0.1 *	1.0 ± 0.3	0.5 ± 0.1 *
*flaK*	Polar flagellar regulator	1.0 ± 0.1	ND	1.0 ± 0.2	ND
*lafA*	Lateral flagellar flagellin	1.0 ± 0.0	1.2 ± 0.2	NT	NT
*lafK*	Lateral flagellar regulator	1.0 ± 0.1	1.1 ± 0.2	NT	NT
*vpsR*	Main activator of VPS production	1.1 ± 0.5	0.2 ± 0.1 **	1.0 ± 0.3	0.3 ± 0.1 **
*vpsT*	Secondary activator of VPS production	1.0 ± 0.3	0.1 ± 0.0 ***	1.0 ± 0.3	0.1 ± 0.0 ***
*vhp*	metalloprotease	1.0 ± 0.4	0.4 ± 0.0 **	1.0 ± 0.3	0.4 ± 0.1 **
*pirA*	AHPND toxin gene	NT	NT	1.0 ± 0.2	0.9 ± 0.2
*pirB*	AHPND toxin gene	NT	NT	1.0 ± 0.3	0.8 ± 0.3

^1^ For each gene, the mRNA level in untreated *V. campbellii* was set at 1 and the levels in the Qstatin-treated cultures were normalized accordingly using the 2^−ΔΔCT^ method. The RNA polymerase A subunit gene (*rpoA*) was used as an internal control. Asterisks indicate significant differences when compared to non-treated *V. campbellii* (independent samples *t*-test; * *p* < 0.05, ** *p* < 0.01, *** *p* < 0.001). ND: no amplification detected. NT: not tested.

## Data Availability

The original contributions presented in the study are included in the article, and further inquiries can be directed to the corresponding authors.

## Abbreviations

The following abbreviations are used in this manuscript:

*V.*	*Vibrio*
AHPND	Acute Hepatopancreatic Necrosis Disease
WOAH	World Organization for Animal Health
LB_35_	Luria–Bertani Medium Containing 35 g/L NaCl
